# Correlation analysis of potential factors influencing graft maturity based on MRI after lateral meniscal allograft transplantation

**DOI:** 10.1038/s41598-020-68153-5

**Published:** 2020-07-09

**Authors:** Shiyou Ren, Xintao Zhang, Xiurong Yu, Ri Zhou, Lu Xu, Zhenglong Lin, Wentao Zhang

**Affiliations:** 1 Department of Sports Medicine and Rehabilitation, Peking University Shenzhen Hospital, 1120 Lianhua Road, Futian District, Shenzhen, 518036 Guangdong China; 2 Anesthesia Operation Center, Hainan Hospital of PLA General Hospital, Sanya, 572013 Hainan China; 3 Radiology Department, Peking University Shenzhen Hospital, Shenzhen, 518036 China

**Keywords:** Orthopaedics, Medical research

## Abstract

The aim of this respective study was to assess the graft signal/noise quotient (SNQ) value and associated factors based on magnetic resonance imaging (MRI) after lateral meniscal allograft transplantation (LMAT). Patients with LMAT were included. The SNQ, width of the anterior horn (WAH), width of the midbody (WMB), width of the posterior horn (WPH) of each lateral meniscus, coronal graft extrusion (CGE), the anterior cartilage meniscus distance (ACMD) and the posterior cartilage meniscus distance (PCMD) were measured using MRI and tested by multivariate stepwise regression analysis. The relative percentage of extrusion (PRE) was calculated. Seventy-one male patients were examined, and 7 patients were lost to follow-up. The SNQ of the meniscus increased from immediately after surgery to 6 months postoperatively, decreased from 6 to 12 months, increased from 12 to 24 months, and increased from 24 to 36 months. The mean SNQ had a significant negative association with the WPH and CGE at 6 months (p < 0.05), the WPH at 1 year (p < 0.05), the PRE of CGE (CPRE) at 2 years (p < 0.05), and the PCMD, CPRE, and PRE of the PCMD (PPRE) at 3 years (p < 0.01) postoperatively. Multivariate stepwise regression analysis showed that the WPH at 6 months, WPH at 1 year, WMD and PCMD at 2 years, and WMD, ACMD and CGE at 3 years were significant independent factors correlated with the mean SNQ of grafts in different periods. Maturation of meniscal grafts fluctuated with time. The maturation process occupied the main role before 1 year postoperatively, but after the maturation process, tearing of the meniscal allograft played the leading role. Changes in an allograft’s location had an obvious association with the SNQ. The WPH influenced the graft SNQ value at 6 months and 1 year postoperatively, but after the maturation process, the WMB and graft extrusion played the same roles.

## Introduction

Fibrocartilaginous menisci play an essential role in the complex biomechanics of the knee joint, such as load distribution, joint stability, articular cartilage lubrication and shock absorption^1^. Acute meniscal tears are the second most common athletic knee injury following anterior cruciate ligament (ACL) tears^2^. For irreparable meniscal injury, meniscectomy is a common surgery. The long-term results of total meniscectomy have been shown to be closely associated with rapid degenerative changes and decreased clinical function and activity levels^3^. In the late 1980s, clinical meniscal allograft transplantation (MAT) emerged as a logical approach to provide pain relief and functional improvement of the knee joint^4^. Compared to meniscectomized knee joints, MAT increases the contact area and decreases the contact pressure on the cartilage surface^5^.

The increased number of MATs has led to the need for better postoperative evaluation of the reconstructed graft. The International Meniscus Reconstruction Experts Forum (IMREF) does not recommend routine second-look arthroscopy after MAT^6^. Magnetic resonance imaging (MRI) is commonly used as a noninvasive tool to evaluate graft status after implantation. The sagittal allograft position, morphologic features, shrinkage, extrusion, intrameniscal signal intensities and degeneration of adjacent articular cartilage are often measured^7–9^. However, few studies have investigated intrameniscal signal intensities after MAT, and their clinical relevance remains unknown^6^.

In addition, an increasing number of studies on the failure rate of MAT have been conducted^10–13^. Potential risk factors influencing the failure incidence of MAT, including graft placement, shrinkage, extrusion, and degeneration of adjacent articular cartilage, have been investigated^7–11^. The presence of severe cartilage damage at the time of MAT can cause an increased failure rate^11^. However, little is known about the correlations of these factors with the maturity of transplanted meniscal allografts^6^.

The purpose of this study was to identify factors that influence graft maturity after lateral MAT (LMAT) based on 3.0-T MRI. We hypothesized that graft maturity is critical in the prevention and management of graft failure, and that some factors based on MRI are significantly correlated with increased graft maturity after LMAT.

## Results

At the final follow-up time point, 7 cases had been lost. No infection or synovitis was observed in any of the participants. However, one case of treatment failure and one case of meniscus allograft dislocation were noted, which were treated with a second operation for meniscal restoration.

**Figure 1:**
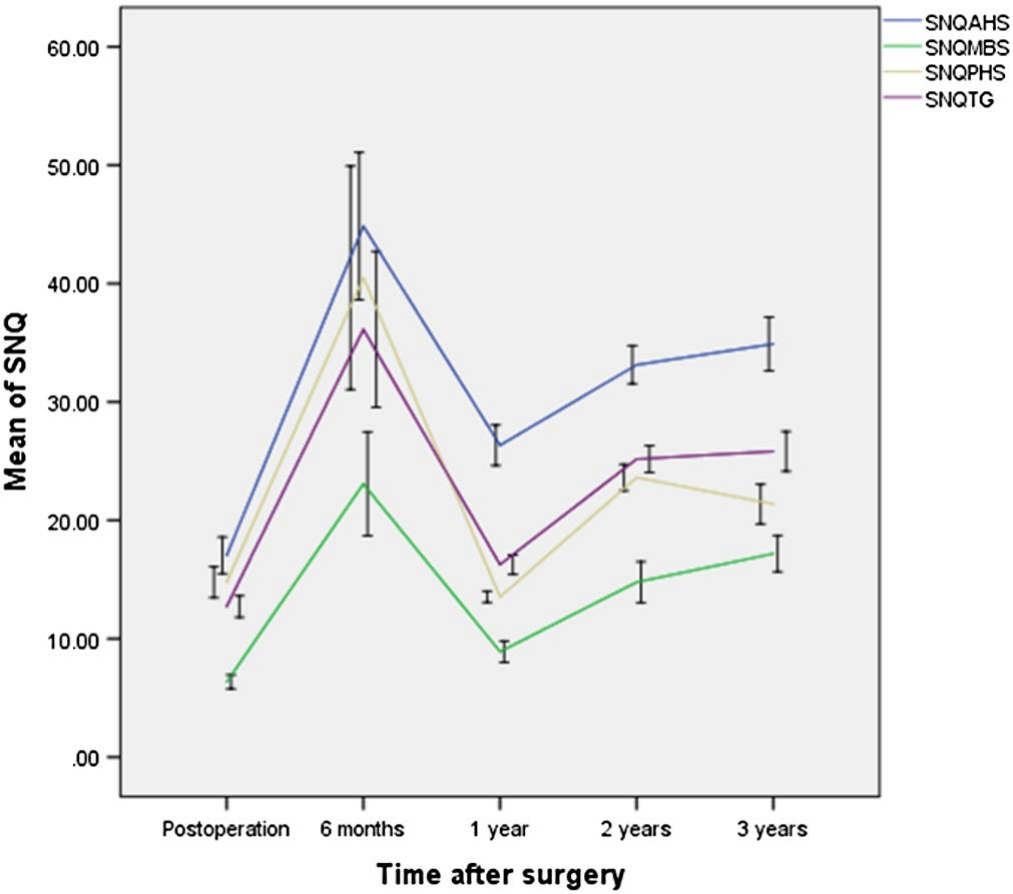
The signal/noise quotients (SNQs) of the total graft and different regions of interest of meniscal allografts in the cohort. SNQ Signal/noise quotient, AHS Anterior horn site, MBS Midbody site, PHS Posterior horn site, TG Total graft.

The intraclass correlation coefficients (ICCs) of intraobserver reliabilities for the signal/noise quotients (SNQs) of the grafts, which were calculated immediately and at 6 months, 1 year, 2 years and 3 years postoperatively, were 0.836, 0.878, 0.827, 0.826 and 0.951, respectively. The ICC of interobserver reliability for the graft SNQ value in the overall sample was 0.77. Generally, the mean SNQ of the meniscal grafts increased from immediately after surgery to 6 months postoperatively, decreased from 6 to 12 months, then increased from 12 to 24 months, and increased slowly from 24 to 36 months. In addition, the peak SNQ occurred at 6 months postoperatively, and the lowest SNQ occurred at 12 months postoperatively. The SNQ values of the anterior horn site, midbody site, and posterior horn site had similar curves. The only difference was that the SNQ of the posterior horn site decreased slowly from 24 to 36 months (Fig. 1).

**Figure 2:**
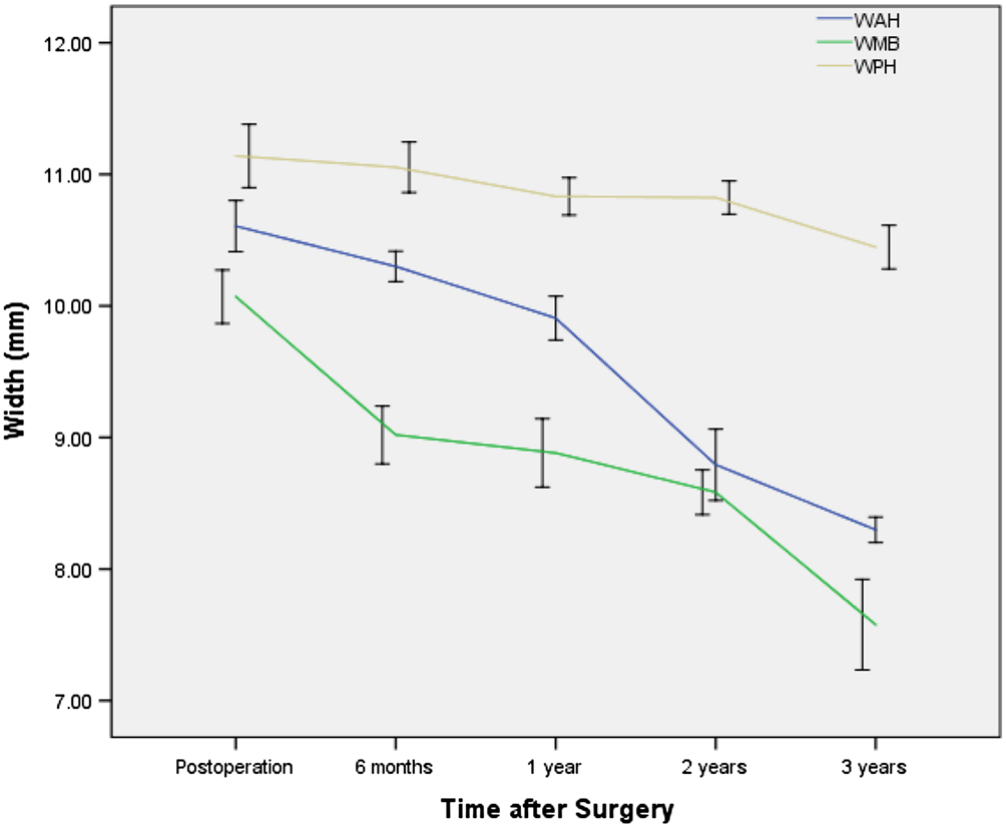
The widths of the anterior horn, midbody and posterior horn of each lateral meniscus with time. WAH Width of the anterior horn, WMB Width of the midbody, WPH Width of the posterior horn.

**Figure 3:**
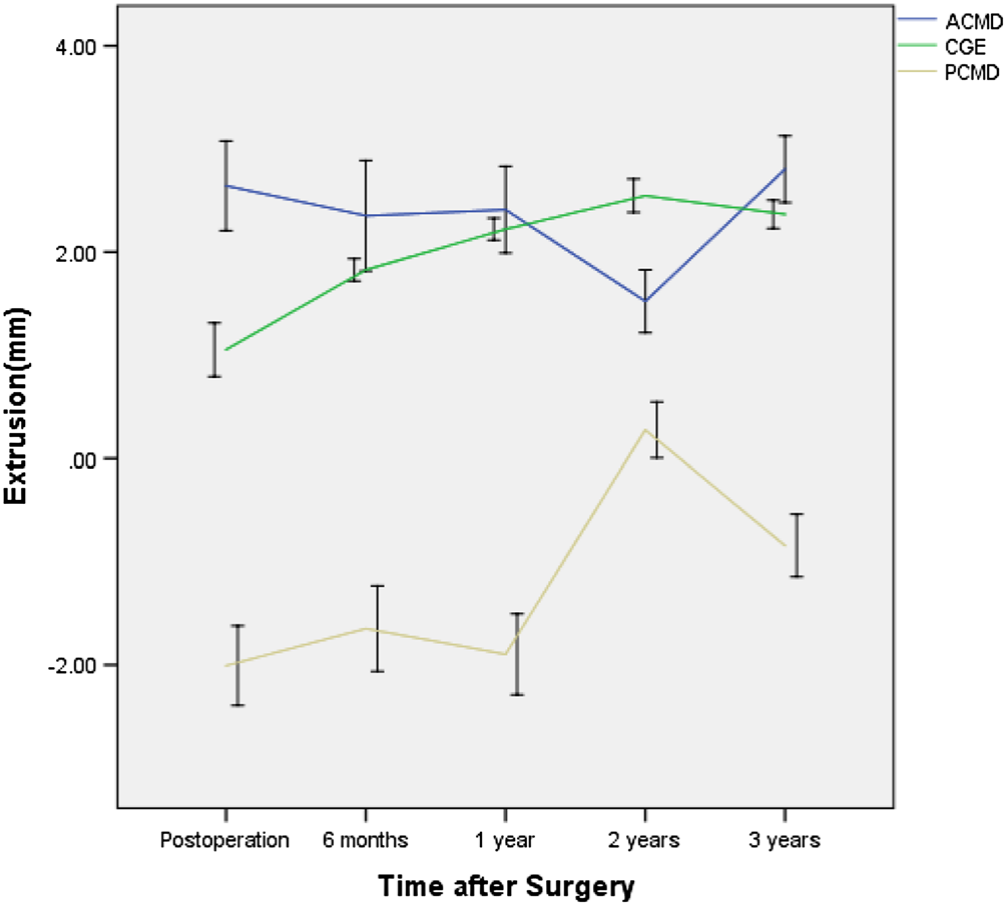
Sagittal and coronal extrusion of the lateral meniscus with time. 0, edge of the tibial margin; +, over the edge of the tibial margin; –, toward the center of the tibial plateau; CGE coronal graft extrusion, ACMD Anterior cartilage meniscus distance, PCMD Posterior cartilage meniscus distance.

**Figure 4:**
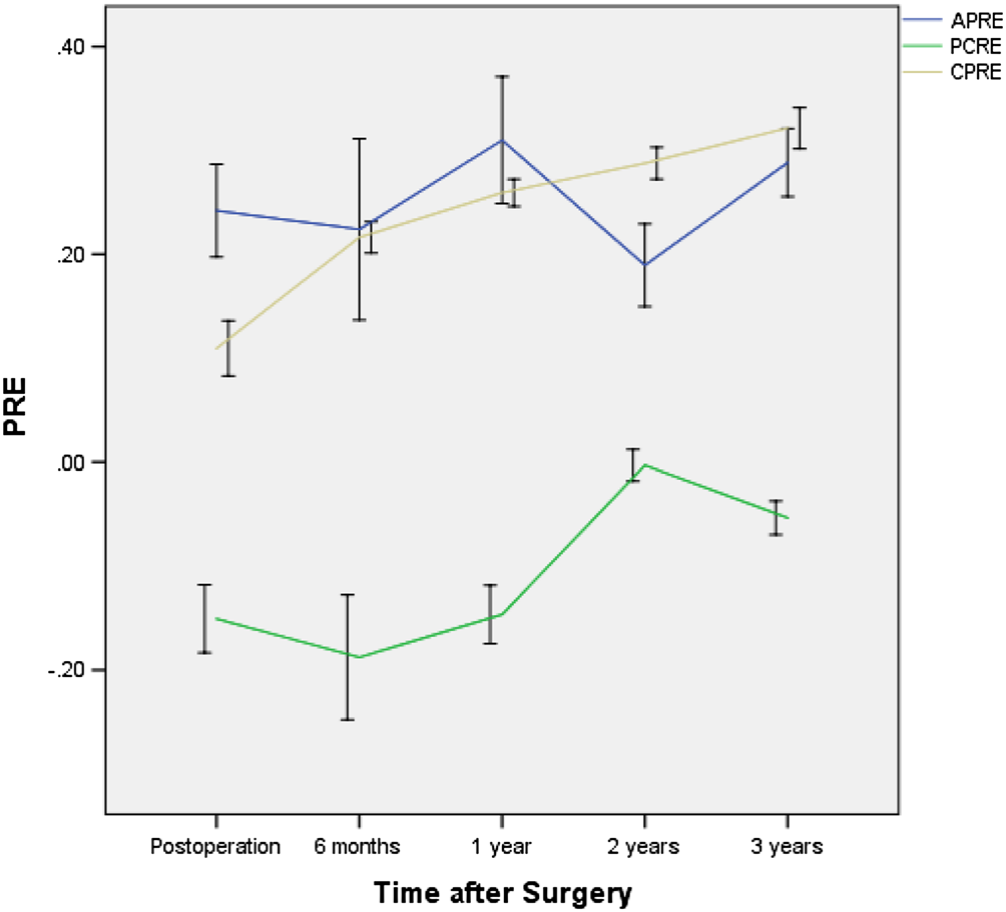
Sagittal and coronal relative percentages of extrusion with time. 0, edge of the tibial margin; +, over the edge of the tibial margin; –, toward the center of the tibial plateau; CPRE Relative percentage of extrusion of CGE, APRE Relative percentage of extrusion of the ACMD, PPRE Relative percentage of extrusion of the PCMD.

The widths of the anterior horn (WAH), midbody (WMB) and posterior horn (WPH) of each lateral meniscus decreased with time (p < 0.05) (Fig. 2), and the WPH changed the slowest, while the WAH changed the fastest. Sagittal and coronal extrusion of the lateral meniscus showed changes according to different curves (Figs. 3 and 4), while coronal graft extrusion (CGE) almost increased with time but became steady after 2 years postoperatively; however, changes in the anterior cartilage meniscus distance (ACMD) and posterior cartilage meniscus distance (PCMD) were more complicated but varied in a contrary manner.

**Figure 5:**
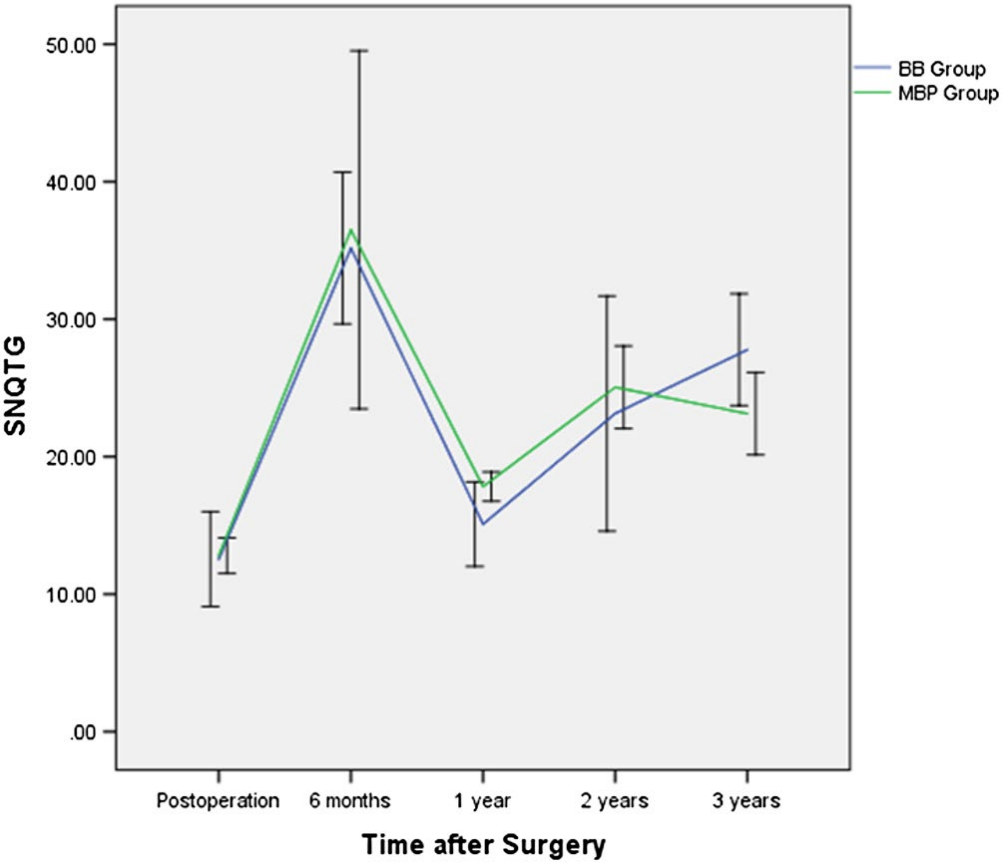
The influence of surgical techniques for LMAT on the signal/noise quotient (SNQ) of the entire meniscal allograft. BBT, Bone bridge technique, MBP Modified bone plug technique, SNQTG SNQ of the total graft.

Similarly, the influence of different surgery techniques was also analyzed. From postoperation to 24 months after surgery, SNQs after LMAT with different surgical techniques had similar curves, and no obvious difference was observed between the 2 groups immediately after surgery or at 6 months, 1 year, and 2 years after surgery (P > 0.05), but at 3 years after surgery, the SNQ after LMAT with the bone bridge technique was obviously higher than after LMAT with the modified bone plug technique (Fig. 5).

**Figure 6:**
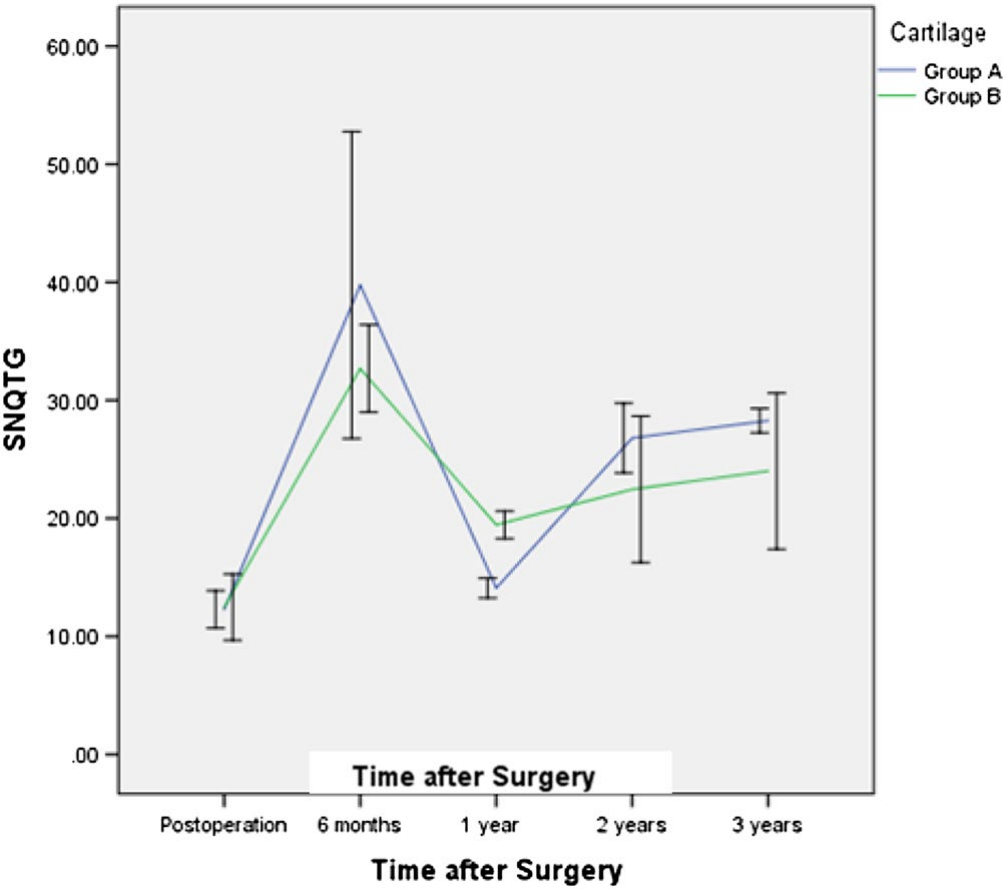
The influence of the cartilage status of the involved compartment before surgery on the signal/noise quotient (SNQ) of the entire meniscal allograft. Participants with Grade O, Grade I, or Grade II cartilage lesions were recorded as Group A, and participants with Grade III or Grade IV cartilage lesions were recorded as Group B; SNQTG, SNQ of the total graft.

Cartilage degeneration of the femoral condyle and tibial plateau of the involved compartment before surgery was evaluated by MRI and graded separately as described by Yulish, with slight modifications^1^. Participants with Grade O, Grade I, or Grade II cartilage lesions were recorded as Group A, and participants with Grade III or Grade IV cartilage lesions were recorded as Group B. The mean SNQs of the meniscal grafts in the two groups changed with time and followed different curves; the SNQ in Group A changed more quickly than that in Group B and had higher peak and lower bottommost values (Fig. 6).

The mean SNQ values at various sites and the mean SNQ value of the total graft in Table 1 reflect the mean signal intensity (SI). Possible associations between several factors (WAH, WMB, WPH, CGE, the relative percentage of extrusion [PRE] of CGE [CPRE], ACMD, the relative percentage of extrusion of the ACMD [APRE], PCMD, and the PRE of the PCMD [PPRE]) and the SNQ values of the grafts are shown in Table 2. The mean SNQ of the grafts had significant negative associations with the WPH and CGE at 6 months postoperatively (p < 0.05), the WPH at 1 year postoperatively (p < 0.05), the CPRE at 2 years postoperatively (p < 0.05), and the PCMD, CPRE, and PPRE at 3 years postoperatively (p < 0.01). However, the mean SNQ had significant positive associations with CGE, the PCMD, the CPRE, and the PPRE at 1 year postoperatively and the PCMD at 2 years postoperatively.

**Table 1:** Possible associations between several factors and the SNQ values of the total grafts.

Variate	SNQ of the AHS	SNQ of the MBS	SNQ of the PHS	Mean SNQ
**6 months**				
WAH	n.s	n.s	n.s	n.s
WMB	n.s	n.s	n.s	n.s
WPH	Spearman = − 0.288, P = 0.039	n.s	Spearman = − 0.279, P = 0.045	Spearman = − 0.282, P = 0.043
ACMD	n.s	n.s	n.s	n.s
CGE	n.s	Spearman = − 0.286, P = 0.04	Spearman = − 0.290, P = 0.037	Spearman = − 0.286, P = 0.040
PCMD	n.s	n.s	n.s	n.s
APRE	n.s	n.s	n.s	n.s
CPRE	n.s	n.s	Spearman = − 0.281, P = 0.044	n.s
PPRE	n.s	n.s	n.s	n.s
**1 year**				
WAH	n.s	n.s	n.s	n.s
WMB	n.s	Spearman = − 0.419, P = 0.001	Spearman = − 0.268, P = 0.046	n.s
WPH	Spearman = − 0.649, P = 0.000	n.s	n.s	Spearman = − 0.501, P = 0.000
ACMD	n.s	n.s	Spearman = − 0.318, P = 0.017	n.s
CGE	Spearman = 0.543, P = 0.000	n.s	Spearman = 0.350, P = 0.008	Spearman = 0.4631, P = 0.000
PCMD	Spearman = 0.505, P = 0.000	n.s	Spearman = − 0.469, P = 0.000	Spearman = 0.509, P = 0.0000
APRE	n.s	Spearman = 0.303, P = 0.023	n.s	n.s
CPRE	Spearman = − 0.596, P = 0.000	Spearman = 0.280, P = 0.045	Spearman = 0.415, P = 0.001	Spearman = 0.584, P = 0.000
PPRE	Spearman = 0.507, P = 0.000	n.s	Spearman = − 0.524, P = 0.000	Spearman = 0.542, P = 0.000
**2 years**				
WAH	n.s	Spearman = − 0.287, P = 0.032	n.s	n.s
WMB	n.s	n.s	n.s	n.s
WPH	Spearman = 0.284, P = 0.034	n.s	n.s	n.s
ACMD	n.s	Spearman = 0.338, P = 0.011	Spearman = − 0.344, P = 0.009	n.s
CGE	n.s	n.s	n.s	n.s
PCMD	Spearman = − 0.477, P = 0.000	n.s	n.s	Spearman = − 0.270, P = 0.044
APRE	n.s	Spearman = 0.521, P = 0.000	Spearman = − 0.272, P = 0.043	n.s
CPRE	n.s	n.s	n.s	Spearman = − 0.303, P = 0.023
PPRE	Spearman = 0.407, P = 0.002	n.s	n.s	n.s
**3 years**				
WAH	n.s	n.s	n.s	n.s
WMB	n.s	Spearman = 0.269, P = 0.045	n.s	n.s
WPH	n.s	n.s	n.s	n.s
ACMD	Spearman = − 0.382, P = 0.004	n.s	n.s	n.s
CGE	Spearman = − 0.322, P = 0.016	n.s	n.s	n.s
PCMD	Spearman = − 0.467, P = 0.000	n.s	Spearman = − 0.282, P = 0.035	Spearman = − 0.327, P = 0.014
APRE	Spearman = − 0.447, P = 0.001	n.s	n.s	n.s
CPRE	Spearman = − 0.471, P = 0.000	Spearman = − 0.313, P = 0.019	Spearman = − 0.381, P = 0.004	Spearman = − 0.433, P = 0.001
PPRE	Spearman = − 0.484, P = 0.000	n.s	Spearman = − 0.303, P = 0.023	Spearman = − 0.337, P = 0.011

*SNQ* signal/noise quotient, *n.s.* no significant difference, *AHS* anterior horn site, *MDS* midbody site, *PHS* posterior horn site, *WAH* width of the anterior horn, *WMB* width of the midbody, *WPH* width of the posterior horn, *CGE* coronal graft extrusion, *CPRE* relative percentage of extrusion of CGE, *ACMD* anterior cartilage meniscus distance, *APRE* relative percentage of extrusion of the ACMD, *PCMD* posterior cartilage meniscus distance, *PPRE* RELATIVE percentage of extrusion of the PCMD.

**Table 2:** Multivariate stepwise regression analysis of SNQ values of the total grafts.

Variable	Regression coefficient	SE	Standard regression coefficient	t	P value
**6 months**					
WAH	13.356	8.599	0.235	1.553	0.127
WMB	− 1.896	5.560	− 0.063	− 0.338	0.737
WPH	− 13.358	5.920	− 0.389	− 2.256	0.029
ACMD	− 0.664	2.529	− 0.054	− 0.263	0.794
CGE	− 14.496	9.693	− 0.240	− 1.496	0.142
PCMD	− 3.060	2.316	− 0.193	− 1.321	0.193
**1 year**					
WAH	0.297	0.754	0.060	0.394	0.695
WMB	− 0.805	0.621	− 0.255	− 1.296	0.201
WPH	− 2.146	0.925	− 0.371	− 2.320	0.025
ACMD	− 0.394	0.411	− 0.201	− 0.958	0.343
CGE	0.403	1.424	0.052	0.283	0.779
PCMD	− 0.597	0.374	− 0.285	− 1.598	0.116
**2 years**					
WAH	− 2.673	1.437	− 0.636	− 1.861	0.069
WMB	2.648	1.200	0.395	2.206	0.032
WPH	− 1.163	2.419	− 0.130	− 0.481	0.633
ACMD	0.464	0.457	0.124	1.015	0.315
CGE	2.499	3.806	0.354	0.657	0.515
PCMD	− 3.380	1.449	− 0.804	− 2.334	0.024
**3 years**					
WAH	− 1.616	2.110	− 0.093	− 0.766	0.447
WMB	3.323	0.755	0.680	4.404	0.000
WPH	− 1.563	1.257	− 0.153	− 1.244	0.220
ACMD	− 1.945	0.728	− 0.375	− 2.671	0.010
CGE	− 3.934	1.884	0.322	− 2.088	0.042
PCMD	1.284	0.800	0.231	1.605	0.115

*WAH* width of the anterior horn, *WMB* width of the midbody, *WPH* width of the posterior horn, *CGE* coronal graft extrusion, *ACMD* Anterior cartilage meniscus distance, *PCMD* posterior cartilage meniscus distance.

Multivariate stepwise regression analysis (Table 2) showed that the WPH at 6 months, the WPH at 1 year, the WMD and PCMD at 2 years, and the WMD, ACMD and CGE at 3 years were significant independent factors correlated with the graft SNQ value at different periods after surgery (β = − 13.358 [WPH at 6 months], 2.146 [WPH at 1 year], 2.648 [WMB at 2 years], 3.380 [PCMD at 2 years], 3.323 [WMB at 3 years], 1.945 [ACMD at 3 years], and 3.934 [CGE at 3 years]; p < 0.05).

## Discussion

The purpose of study was to clarify the intrameniscal signal intensity after MAT that represents graft maturity. However, only studies on graft signal intensity after ACL repair have been published, which might clarify the implications of intrameniscal signal intensities after MAT. Graft strength was quantitatively analyzed by measuring graft signal intensity on MRI^14–16^. Graft signal intensity has a significant negative linear correlation with graft strength^17,18^. High signal intensity on MRI indicates a decrease in the mechanical properties of the reconstructed graft. The SNQ represents graft maturity based on MRI images^19^: High graft signal intensity corresponds to a high SNQ value and signifies inferior graft maturity^20^. This effect was the same after MAT, but our study found that a high SNQ value also represents destruction, especially after the graft maturation process. Graft maturation varies considerably depending on the time interval after surgery. The mean SNQ values of LMAT grafts increased from immediately after surgery to 6 months postoperatively, decreased from 6 months to 1 year postoperatively, and increased slowly between 1 and 3 years postoperatively because tearing of the meniscal allograft played a leading role after the maturation process.

The SNQ value had some associations with different parts of grafts. Previously, Zaffagnini et al.^15^ showed that most cases with collagen meniscus implants had similar SI curves that subsequently fluctuated with time, while the SIs of allografts started increasing at 6 months, peaked after 1 year, decreased between 1 and 5 years, and increased slowly from 5 to 10 years. This finding is slightly different from ours. The reasons for this discrepancy may be that the allograft and collagen implants entered the remodeling process after implantation, the grafts underwent revascularization and resynovialization but at different paces, and the maturation of meniscal allografts was faster; 1 year after surgery was a critical period for full maturation of the lateral meniscal allografts.

Regarding the distribution of the SNQ after LMAT, we found that the AHS always had a higher SNQ value—SNQ of AHS > SNQ of PHS > SNQ of MBS. However, McCulloch et al.^21^ found that the native meniscus under tibiofemoral contact pressures was evenly distributed over the anterior and central portions of the plateau, with the highest peak pressures observed posteriorly, and the nonanatomic transplant state and anatomic state both displayed greater peak pressures as the load was distributed over a smaller area but were shifted to the anterior portion of the plateau. The shift of the load anteriorly may be the reason why the SNQ value of the AHS was the greatest.

In addition, we found that the widths of the anterior horn, midbody and posterior horn of each lateral meniscus decreased with time, but the WPH changed the slowest, the WMB decreased quickly in the early healing period, and the WAH decreased quickly in the midterm period. The study by Lee et al.^8^ showed a similar conclusion for shrinkage of the WPH and WMB, but no data for the WAH were available in his study. However, in the study by Kim et al.^22^, no significant progression of shrinkage was observed between 3 and 12 months postoperatively. The reason for this effect might be that in Kim’s study, shrinkage was calculated by multiplying the width and height of the three sections and adding these values together. We also found that the widths of the allograft showed an obvious significant negative association with the SNQ value of the graft at 1 year postoperatively, and the allograft was wider. Additionally, the allograft presented better revascularization, and the area without revascularization dissolved. However, in 2 years and 3 years, the association between the widths (WPH and WMD) of the allograft and its SNQ value was positive, indicating that the intrameniscal SI is higher in cases with wider allografts. This effect might be caused by a wider graft bearing more load between the tibial plateau and femoral condyle. This stress causes variations in the SNQ value. However, until recently, relevant literature to confirm our speculation was lacking.

In our study, CGE and the CPRE increased with time but became steady after 2 years postoperatively; however, changes in the ACMD, APRE, PCMD and PCRE were more complicated but varied in a contrary manner, indicating that the sagittal locations of allografts were constantly changing. A study by Kim et al.^22^ showed that in the sagittal plane, the mean absolute ACMDs were 2.59 ± 1.75, 2.58 ± 1.85, and 2.37 ± 1.60 mm, and the mean relative ACMDs were 20.7% ± 13.1%, 20.6% ± 13.8%, and 19.0% ± 12.2% at the 3 follow-up time points. The mean absolute PCMDs were − 1.23 ± 3.34, − 1.28 ± 3.08, and − 1.42 ± 2.77 mm, and the mean relative PCMDs were − 10.3% ± 25.9%, − 11.0% ± 24.6%, and − 12.2% ± 23.2% at the same time points. In the coronal plane, the mean absolute meniscal extrusion values at 6 weeks, 1 year, and the final follow-up were 2.90 ± 0.94, 2.85 ± 0.97, and 2.83 ± 0.89 mm, respectively, and the mean PREs were 27.0% ± 9.4%, 27.1% ± 10.1%, and 27.8% ± 9.7%, respectively. These findings suggest that extrusion of a meniscal allograft in either the coronal or sagittal plane does not progress after LMAT, which differs from our results. In our study, extrusion of meniscal allografts in either the coronal or sagittal plane obviously changed with time; the anterior horn and the posterior horn both moved anteriorly because they belong to an integral unit. These areas had synergic movement similar to that in the studies by Rankin et al.^23^ and Scholes et al.^24^

In addition, the correlations of the SNQ values of the grafts with the ACMD, APRE, CGE, CPRE, PCMD and PPRE were very complicated, indicating changes in the allografts’ locations, and our study showed that location changes have an obvious association with the SNQ after the maturation process (at 2 years and 3 years postoperatively) (Tables 1 and 2). If a graft has greater CGE or a higher CPRE, then the graft bears less load and has a better bloody supply. Our data confirmed that CGE and the CPRE had negative associations with the SNQ of the MBS and the mean SNQ of the grafts at 6 months but positive associations at 1 year, indicating that greater CGE or a higher CPRE was better for vascularization at 6 months; however, at 1 year, greater CGE or a higher CPRE indicated slow organizational dehydration, while less CGE or a lower CPRE reflected better tissue maturation. These results are consistent with those of some laboratory studies showing that the mechanical environment of load-bearing tissues demonstrates better maturation^25,26^. After meniscal maturation, CGE and the CPRE had negative associations with the SNQ of the MBS and the mean SNQ of the grafts at 2 years and 3 years. Movement toward the center of the tibial plateau corresponded to more load-bearing, degeneration and abrasion, and additionally, a higher SNQ value was observed.

The SNQ changed more quickly in the modified bone plug technique (MBP) group than in the bone bridge technique (BBT) group and had similar peak and lower bottommost values. At 2 years and 3 years postoperatively, the SNQ in the BBT group was higher, showing that better cartilage was good for vascularization at 6 months and for faster organizational dehydration and better tissue maturation at 1 year. The cases with a worse cartilage status showed lower SNQ values of the grafts after the maturation process. Previously, Saltzman et al.^27^ compared a patient series with full-thickness chondral defects who underwent MAT with a patient series with no chondral defects and found no differences in changes in individual patient-reported outcomes (PROs) from the preoperative period to the final follow-up period. However, Kempshall et al.^28^ showed that outcome measures after MAT in cases with minimal chondral damage continued to improve at 2 and 3 years postoperatively, with slightly greater improvement than that in cases with significant chondral damage.

## Limitations

Only some factors based on MRI were analyzed in this study. Whether other factors influence graft maturation, such as clinical outcomes and lower limb power lines, is unclear. Only males were recruited in this study. No females were included to avoid any influence of differences in hormone levels on graft maturity. Therefore, our results are representative of only some patients and not the entire population. Future studies in this field will provide valuable information regarding sex differences and the influence of other factors on graft maturity. Only 64 LMAT cases were ultimately investigated, and the number of studies was small. A larger sample size for further investigations should be utilized in the future. Finally, this study was limited by our use of the SNQs of some graft sites to represent the SNQ of the entire graft, and this method might have limitations.

## Methods

This retrospective study was performed after ethical approval was received from the ethics committee of Peking University Shenzhen Hospital. All methods were carried out in accordance with relevant guidelines and regulations. All subjects included provided informed consent. From January 2008 to December 2015, male patients who underwent unilateral MAT by the same senior doctor were included. Females were excluded due to concerns about different hormonal levels, which might influence graft maturity^20^. More than 3 years of follow-up and assurance of the uniformity of surgery-related factors were necessary. The inclusion criteria were as follows: (1) unilateral MAT, (2) no history of reinjury of the affected knee, and (3) 18–50 years of age. Participants were excluded if they had osteoarthritis (confirmed by surgery), combined ligament injuries, or a severe synovial reaction at the time of surgery.

**Figure 7:**
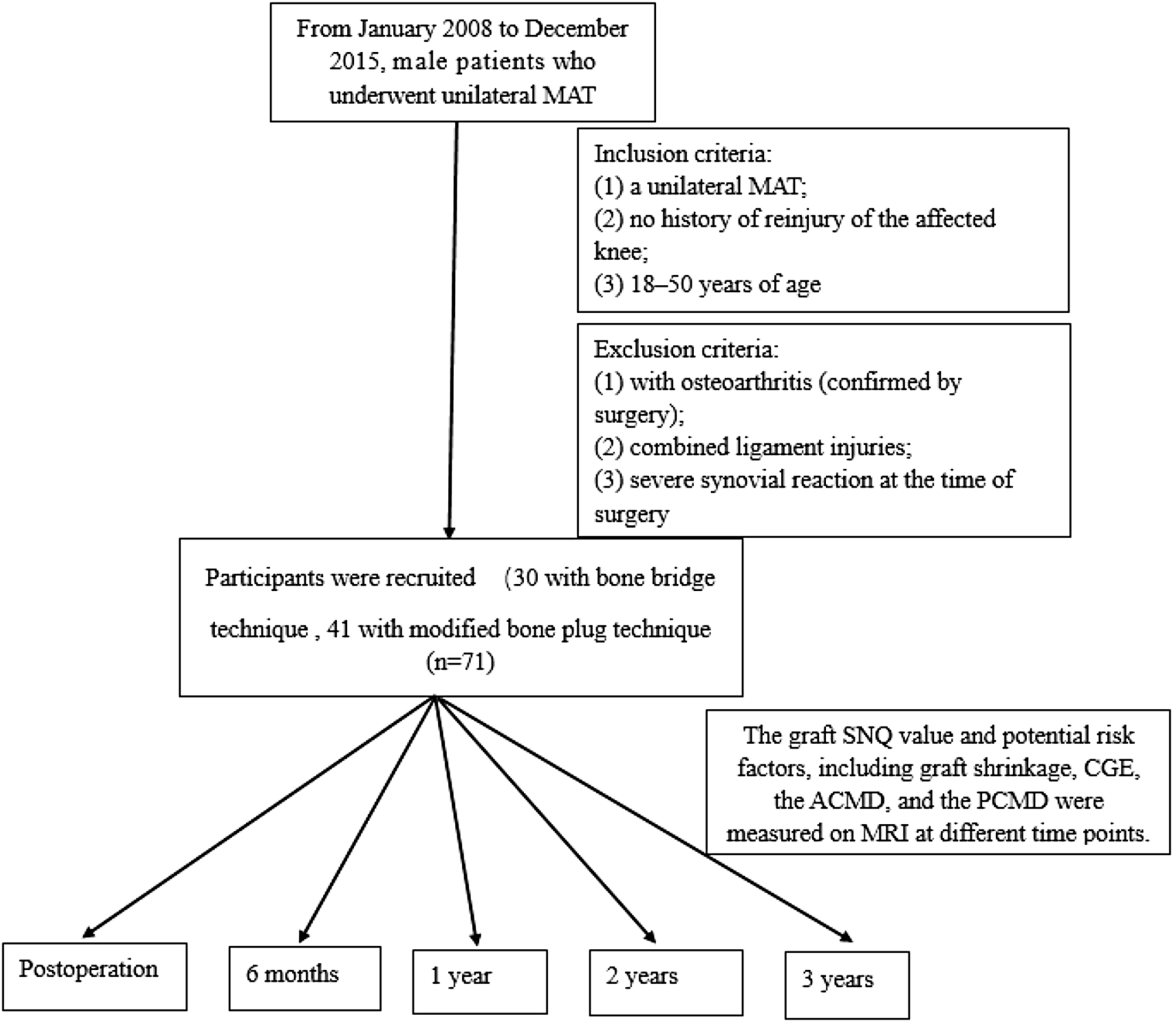
Flow chart of data collection.

A total of 71 patients were recruited for this study (Fig. 7), including 30 patients who underwent the bone bridge technique^29^ (BBT group) and 41 patients who underwent the modified bone plug technique^30^ (MBP group). After surgery, all patients followed the same rehabilitation principles: Early range of motion (ROM) movements in the range of 0° to 60° for 15 min daily were encouraged beginning 1 week postoperatively to minimize the deleterious effects of immobilization; gradual progression to full weight-bearing was permitted by 6 weeks postoperatively; a double upright hinged brace was used during the designated protection phase; flexion was limited to 90° for 14 weeks postoperatively; gentle jogging was allowed between 4 and 6 months, but running was not advised before 6 months postoperatively; and finally, a return to sports was anticipated between 6 and 9 months^30^. The patient demographic data are shown in Table 3. The two groups did not differ significantly in age (p > 0.05) or body mass index (BMI) (p > 0.05).

**Table 3:** Participant demographic data of the study groups.

	BBT	MBP	P value
Cases	30	41	–
Age, mean ± SD, years	33.2 ± 8.2	31.5 ± 6.1	P = 0.35
Body mass index (kg/m^2^)	25.5 ± 4.1	24.7 ± 5.7	P = 0.52
Operative side, left/right, n	12/18	13/21	P = 0.88
Yulish grade^1^	2.1 ± 1.1	1.7 ± 1.3	P = 0.33

*BBT* bone bridge technique, *MBP* modified bone plug technique, *SNQTG* SNQ of the total graft.

### MRI scan and image analysis

**Figure 8:**
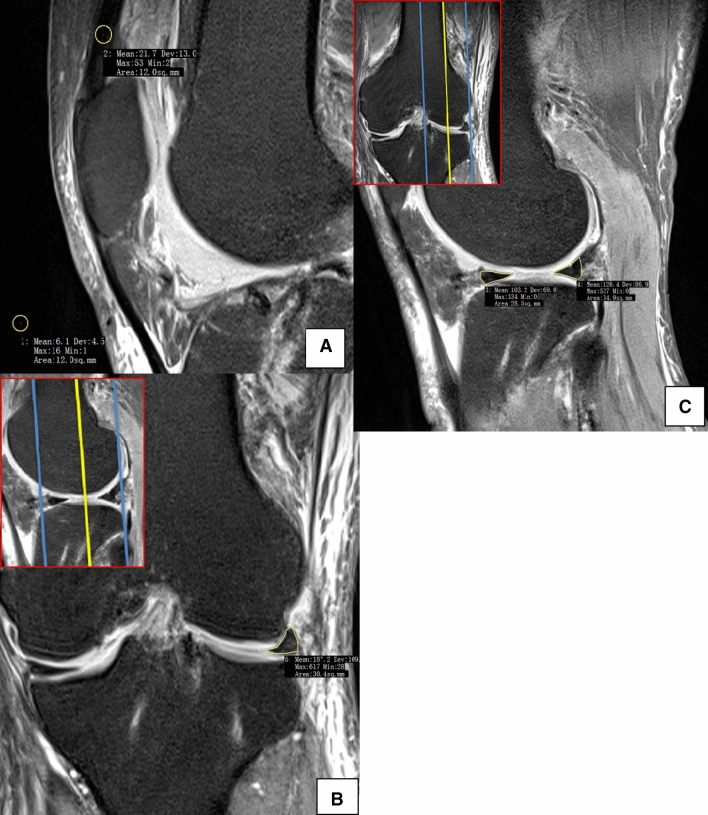
Magnetic resonance image of the knee showing the positions of the 5 regions of interest (area of the circle, 0.10 cm^2^; the polygon of the area depends on the outline drawing of the meniscal allograft), including the (1) background site, (2) quadriceps tendon, (3) anterior horn site, (4) posterior horn site, and (5) midbody site. All measurements were performed on coronal (**b**) and sagittal (**c**) magnetic resonance imaging sections. The midcoronal section was determined as the middle cut among the serial coronal images from the anterior to the posterior meniscocapsular junction of the lateral meniscus; the midsagittal section was selected among the serial sagittal images from the lateral tibial eminence to the lateral meniscocapsular junction of the lateral meniscus in the same manner.

The patients were asked to return for MRI immediately and at 6 months, 1 year, 2 years and 3 years after surgery. All of the participants had at least 1 h of rest before the MRI scan. Then, imaging of the knees was performed in a relaxed extended position with a 3.0-T MRI scanner (MAGNETOM Spectra, A Tim system; Siemens). Sagittal oblique fat-saturated proton density images were obtained as follows: repetition time, 3,000 ms; echo time, 45 ms; flip angle, 160°; matrix, 320 × 320; field of view, 15 × 15 cm; slice thickness, 3 mm; and scan time, 2 min 41 s. The image data were transferred to Siemens workstation software (NUMARIS/4, SyngoMR B17; Siemens) for calculation. All calculations at the midbody of each lateral meniscus were evaluated on midcoronal sections, and those at the posterior and anterior horns were measured on midsagittal sections of T2-weighted images and midcoronal or midsagittal sections, which were determined as the middle cuts among the serial coronal images from the anterior to the posterior meniscocapsular junction of the lateral meniscus and the serial sagittal images from the lateral tibial eminence to the lateral meniscocapsular junction of the lateral meniscus^8^ (Fig. 8b and c).

The SI was calculated at the graft sites as well as two other sites (the quadriceps tendon site at approximately 2 cm at the top of the patella and the background at approximately 2 cm anterior to the patellar tendon) using a region of interest (ROI) (Fig. 8). The mean SI of the meniscal graft was averaged with three sites of graft intensity (the anterior horn site, middle body site, and posterior horn site). To quantify the normalized SI of the meniscal graft, the SNQ for each graft site was calculated using the following equation: SNQ = (signal of the meniscal graft − signal of the quadriceps tendon)/signal of the background. All measurements were taken by two investigators, and repeated measurements were performed on 2 days at least 1 month apart^31^.

In addition, we also measured many other factors, including the following: the WAH, the WMB, the WPH of each lateral meniscus, the grade of cartilage lesions before surgery, CGE, the CPRE, the ACMD (defined as the distance from the anterior articular margin to the anterior border of the anterior horn of the meniscus), anterior graft extrusion, the APRE, the PCMD (defined as the distance from the posterior articular margin to the posterior border of the meniscus), posterior graft extrusion, and the PPRE^1,8,9,32^.

### Statistical analysis

Statistical analysis was performed with SPSS software for Windows (version 23.0, SPSS Inc., Chicago, IL, USA), and the results were reported as the mean and standard deviation. The data were tested for normality using the Shapiro–Wilk test. ICCs were assessed by examining intraobserver reliabilities. The coefficients were interpreted as poor if ICC < 0.4, marginal if 0.4 < ICC < 0.75, and good if ICC > 0.75. Spearman’s correlation coefficients were calculated between the graft SNQ value and potential risk factors, including graft shrinkage, CGE, the grade cartilage lesions before surgery, the ACMD, and the PCMD. Multivariate stepwise regression analysis was performed to further assess the independent factors correlated with the graft SNQ value. The level of significance was set as P < 0.05;β < 0.2, and the expected correlation coefficients were referenced from Li’s et al.^20^ study. Calculation of the sample size yielded 62 patients.

## Conclusion

The SNQ values of meniscal grafts fluctuated with time because the maturation process occupied the main role before 1 year postoperatively, but after the maturation process, tearing of the meniscal allograft played the leading role. LMAT with the MBP resulted in better midterm maturation than LMAT with the BBT. The WPH was a significant independent factor correlated with the graft SNQ value at 6 months and 1 year after surgery, but after maturation of the WMB, graft extrusion was a significant independent predictor of the graft SNQ value.
